# The Fas/FasL System in Reproduction: Survival and Apoptosis

**DOI:** 10.1100/tsw.2002.856

**Published:** 2002-06-29

**Authors:** Gil Mor, Shawn Straszewski, Marijke Kamsteeg

**Affiliations:** Department of Obstetrics and Gynecology, Yale University School of Medicine, New Haven, CT, USA

**Keywords:** Fas, Fas Ligand, estrogen, macrophages, apoptosis

## Abstract

For centuries, the question of “whether there is life after death” has intrigued the mind of philosophers, and the same question fascinates researchers in the field of apoptosis today. The death of a cell is by no means the end of the story. On the contrary, growing evidence suggests that the clearance of apoptotic bodies by macrophages is an important regulatory component in tissue renewal. Without death by apoptosis, the life of reproductive tissues and their function would not be possible. The survival signals that counteract cell death also prepare the cells for apoptosis, and dead cells are important stimuli for tissue survival. The Fas/FasL system is an important mediator in apoptosis and is and excellent example of this apparently contradictory phenomenon.

## INTRODUCTION

Apoptosis, or programmed cell death, is an active process essential for the development and homeostasis of all multicellular organisms[1]. Following cycles of proliferation and differentiation, unnecessary or potentially dangerous cells undergo apoptosis without affecting neighboring cells. As the primary mechanism of physiological cell loss, apoptosis plays an important role in the maintenance of normal tissue function throughout the body[2]. A delicate balance between the factors controlling cell proliferation and those controlling apoptosis properly maintains this tissue homeostasis. Disturbance in this balance may lead to either insufficient or excessive apoptosis which then can contribute to a variety of pathological conditions including cancer, AIDS, and autoimmunity[2].

Due to the cyclic nature of the female reproductive system, imposed by the episodic release of ovarian steroids, female reproductive organs such as the ovary, endometrium, and mammary gland sustain continuous cycles of cell growth and apoptosis in response to hormonal changes, and, therefore, are particularly dependent on this means of cell death. These cycles of cell growth/death, also known as "tissue remodeling", play an important role not only in the normal physiology of the tissue but also in the prevention of neoplastic transformation and cancer formation. Under the influence of survival factors such as estrogen, insulin, or LH/FSH, cells proliferate and increase in number (stage I). Subsequently, hormones such as progesterone or prolactin induce cell differentiation, which conveys two aspects—one is to reduce the rate of cell proliferation and the other to commit differentiated cells to undergo apoptosis at the end of their function (stage II and III, respectively)[3].

However, this picture would be incomplete without mentioning a fourth stage, which is the removal of apoptotic cells (clearance) and its role in tissue homeostasis (Fig. 1).

In this review we will discuss the interaction between sex hormones as survival factors, the Fas/FasL system, one of the main apoptotic pathways, and macrophages, in tissue remodeling of the female reproductive organs and its implication in normal and pathophysiology.

## APOPTOSIS

Apoptosis is a morphologically defined cell death, and can be divided into three sections: initiation, execution, and termination. Unlike necrosis, which is generally caused by physical or chemical

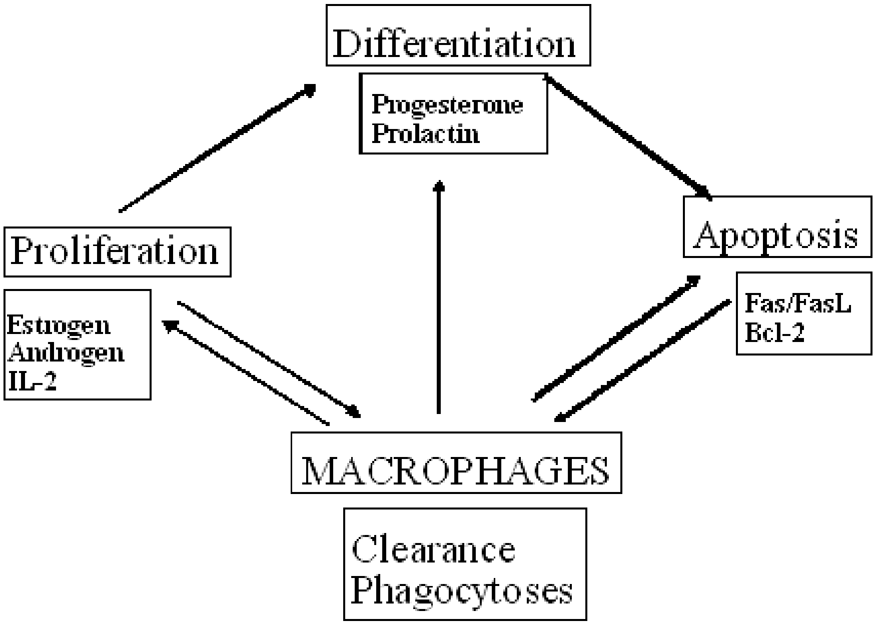

**FIGURE 1**. A complete view of tissue homeostasis. Our understanding of proliferation, differentiation, and cell death is incomplete without acknowledging the role of tissue clearance by macrophages.

injury, apoptosis is normally triggered as a cellular response to survival factor deprivation. Moreover, necrosis results in the release of the cytoplasmic contents of the cell into the extracellular matrix followed by an inflammatory response, whereas apoptosis occurs without activation of the immune system. Apoptosis is typically characterized by membrane inversion, cell shrinkage, chromatin condensation, and DNA degradation[4]. In the terminal stages of apoptosis, the so-called apoptotic bodies that form during the execution phase are quickly engulfed by phagocytic cells such as macrophages[5], thereby preventing lysis and release of intracellular proteins, which otherwise may trigger autoimmune responses (see below).

Apoptosis can be initiated either by external factors including UV- or γ-irradiation, chemotherapeutic drugs, and death receptors (known as the "extrinsic pathway")[6,7], or by internal factors such as DNA damage and p53 activation (the "intrinsic pathway")[8]. In this latter pathway, the death signal is directed to the mitochondria and is regulated mainly by genes of the bcl2 superfamily (reviewed in [9,10]). The proapoptotic Bcl-2 family members, such as Bax and Bak, increase the permeability of the mitochrondrial membrane to cytochrome-c release. Consequently, a macromolecular complex called an apoptosome is formed, which activates caspase-9 with the help of apoptotic protease activating factor (APAF-1). In turn, caspase-9 activates caspase-3 and -7, the point at which the two pathways converge.

If apoptosis is executed through the extrinsic pathway, the death signal is initiated by one of the members of the Tumor Necrosis Factor (TNF) receptor superfamily[11,12]. In order to transduce the apoptotic signal, TNF receptors have intracellular homophilic death domains (DD), which mediate protein–protein interactions with other DD-containing adaptor proteins[13]. To date, six members of this family have been identified and include Fas (APO-1/CD95), TNF-R1, DR3, TRAIL-R1, TRAIL-R2, and DR6[12,14]. Among the death receptors, Fas is the most widely studied and best characterized[11,15]. Analogous to the other TNF family members, Fas is activated by its natural ligand, FasL, which exists in a soluble and membranal form[12].

## THE FAS/FASL SYSTEM

### Structure and Function

The pathway by which signaling through Fas leads to cell death (apoptosis) has been studied extensively[5]. It begins with the binding of FasL or an agonistic Fas monoclonal antibody (in experimental setups) to the extracellular region of the Fas receptor, resulting in its trimerization. Upon receptor oligomerization, the intracellular Fas-associated death domain (FADD) binds to the cytoplasmic tail of Fas via its death effector domain (DED)[13]. In turn, FADD recruits other cellular proteins through DD to form the death-inducing signaling complex (DISC). Once the DISC is assembled, procaspase-8 or -10, the 'initiator' cysteine proteases of the pathway, are able to bind to FADD. Analogous to other proteases, caspase-8 and -10 normally exist in the cytoplasm in their nonactive zymogen form until activated. According to the induced proximity model, high local concentrations of procaspase-8 and -10 are trans- or autocatalytically cleaved once in close proximity to the DISC[16]. This is believed to occur by a two-step cleavage process that results in the formation of active caspase-8 or -10 heterotetramers[17]. Following activation, caspase-8 or -10 initiate the caspase cascade, which terminates with caspase-3 and -7 cleavage. As effector caspases, caspase-3 and -7 cleave a variety of substrates, including DNA repair enzymes and endonucleases such as inhibitor of caspase-activated deoxyribonuclease (ICAD). Consequently, caspase-activated deoxyribonuclease (CAD) is released from ICAD and enters the nucleus to nonspecifically cut the genomic DNA into 200 base-pair fragments, eventually ending in apoptosis[18]. Altogether, this signaling through protein–protein interactions and proteolytic cleavage steps provide several points in the Fas-signaling pathway to modulate apoptosis (see Fig. 2).

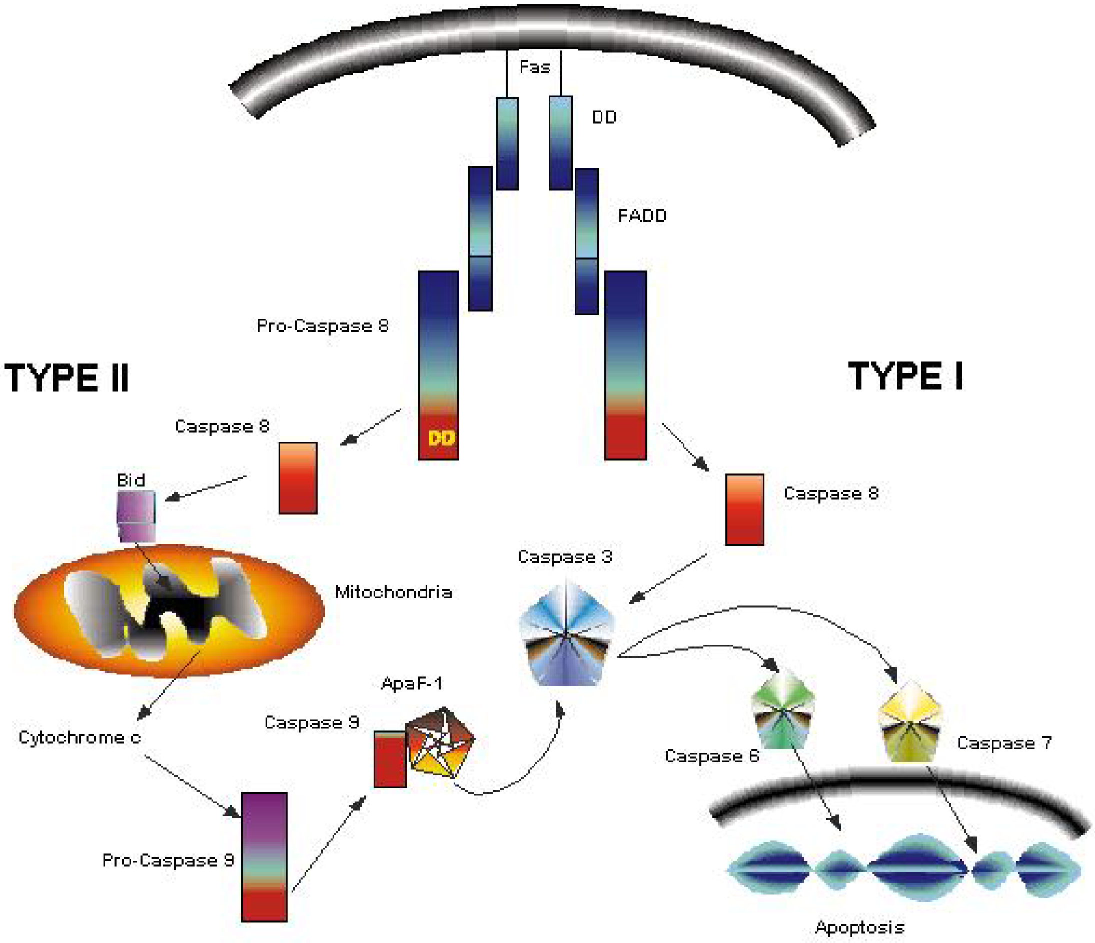

**FIGURE 2**. Schematic representation of the intracellular components of the Fas/FasL pathway.

## Regulation

Since the machinery for Fas-mediated apoptosis is readily present within most cells, several mechanisms exist to keep cell death under control. Regulation of Fas-mediated apoptotic machinery may occur at many steps along the recruitment-activation pathway. At the receptor level, Fas-mediated apoptosis can be blocked by either dominant negative decoy receptors, receptor endocytosis or by soluble FasL (sFasL)[17]. Additionally, several intracellular inhibitors that block the Fas-signaling pathway have been characterized. As proximal inhibitors of the extrinsic pathway, Flice-like inhibiting proteins (FLIPs) are believed to compete with caspase-8 for binding to FADD, thereby preventing apoptotic signaling events downstream caspase-8 activation[5]. The blocking of the Fas-pathway also may occur at the effector stage (casapase-8 and -3 activation) by viral protein products, such as cytokine response modifier A, p35, and inhibitors of apoptosis (IAP)[19].

### *Signaling by Fas: The Two Pathway Model*

What was originally thought to be a discrepancy between the data obtained from independent Bcl-2 overexpression studies and their effect on Fas led to the identification of two cell types termed Type I and Type II[20] (Fig 2). Type I cells can undergo death receptor–mediated apoptosis without mitochrondrial support (extrinsic pathway), whereas type II cells require the release of cytochrome-c from mitochrondria (intrinsic pathway) in order to translate the death signal. In contrast to type II cells, type I cells contain large amounts of DISC and caspase-8. As a consequence, type I cells can rely solely on the extrinsic pathway, whereas type II cells are dependent on both the extrinsic and intrinsic pathways to execute apoptosis. Thus, since Bcl-2 overexpression blocks the intrinsic pathway only, these observations might explain why certain cells are sensitive and others insensitive to Bcl-2 overexpression[21]. Besides cell lineage, the nature of the apoptotic stimulus and the microenvironment (cytokines, growth factors, etc.) are likely to play a role in whether apoptosis is executed by the extrinsic or intrinsic pathway[22]. However, the idea of type I and II is still subject of controversy, some investigators have proposed that that type I or type II cells do not exist but FasL-induced apoptosis in any cell type (being sensitive to this form of apoptosis) occurs as a mixture of both pathways[23].

## THE ROLES OF THE FAS/FASL SYSTEM IN TISSUE HOMEOSTASIS

The Fas/FasL system was originally characterized in lymphocyte homeostasis, during which both immature and mature T cells are eliminated by a Fas-dependent mechanism termed activation-induced cell death (AICD)[24]. In particular, Fas and FasL have been implicated in the clonal deletion of self-reactive thymocytes in secondary lymphoid tissues, cytotoxic T cell killing, and the removal of activated peripheral T cells following an immune response[25,26]. A loss-of-function mutation in the gene encoding either Fas or FasL, as demonstrated in homozygous *lpr* (lymphoproliferation) and *gld* (generalized lymphoproliferation disease) mice, respectively, results in progressive lymphocyte accumulation and severe autoimmunity[27]. Details about the role of the Fas/FasL system on lymphocyte homeostasis have been reviewed extensively (see recent reviews by Kramer[5] and Sharma[28]).

## THE FAS/FASL SYSTEM IN REPRODUCTIVE TISSUES

Reproductive organs such as the endometrium, mammary gland, and ovary undergo cycles of cell proliferation and cell death as a result of changes in hormonal levels. During each cycle there is a massive increase in cell number, which is then decreased by a coordinate process of cell death. New evidence from our laboratory and others indicate the existence of a close interaction between sex hormones, mainly estrogen and progesterone, and the Fas/FasL system in the control and maintenance of tissue homeostasis. We will review work done in the endometrium, mammary gland, and ovary.

### Endometrium

The human endometrium, in preparation for pregnancy, undergoes cell growth in response to estrogen and progesterone. Recent studies have demonstrated the expression of Fas and FasL in human endometrium throughout the menstrual cycle. Using electron microscopy and immunohistochemistry, Yamashita and coinvestigators localized Fas and FasL to the Golgi apparatus and vesicles in the late proliferative phase. In addition, the same study revealed that both Fas and FasL are coexpressed in the plasma membrane of endometrial cells during the secretory phase of the menstrual cycle[29]. This expression pattern suggests that the Fas/FasL system is involved in apoptosis of the human endometrium during menstruation and responds to hormonal changes.

Under the influence of estrogen, endometrial cells in the proliferative phase of the menstrual cycle are stimulated to divide and become resistant to apoptosis. Surprisingly, we found that estrogen increased FasL expression in endometrial cells, thymocytes, and breast cancer cells[25,30,31].

It became clear that estrogen–estrogen receptor complexes directly regulate the FasL expression through the presence of an Estrogen Regulatory Element (ERE) in the FasL promoter and mutations in the ERE region were shown to block the estrogenic effect completely[31,32]. Furthermore, the estrogenic effect on the FasL expression is not only dependent on the ERE but also on the transcription factor AP-1 motif, also present in the FasL promoter[33]. Introduction of mutations in the AP-1 motif reduced the effect of estrogen by 40%. However, the effect of estrogen on FasL expression seems to be dependent on the subtype of the estrogen receptor (ER-α or ER-β) present, since no reduction was observed in cells expressing ERβ alone (Fig. 3). This suggests that only ERa is capable of activating AP-1. Furthermore, we demonstrated that estrogen induces cell proliferation in neuronal-like cells expressing ERa, but induces apoptosis through the Fas/FasL system in those expressing ERβ, which correlates with the loss of ERβ in proliferative and malignant cells[34,35,36]. Since estrogen enhances proliferation and increases FasL-expression, which seems contradictory, it is essential to note that an increase in FasL expression does not necessarily result in an increase in apoptosis, because Fas needs to be expressed as well. Therefore, increase in FasL expression by estrogen while it induces cell proliferation might serve to prepare the cell for apoptosis after proliferation and differentiation and thereby maintaining tissue homeostasis.

During the secretory phase, progesterone inhibits further proliferation and induces terminal differentiation. Through the process of differentiation, cells become again sensitive to apoptosis by the increase in the expression of proapoptotic genes. While estrogen has a regulatory effect on FasL expression it does not affect Fas expression[31]. Contrary to estrogen, progesterone regulates the expression of Fas but not FasL. The induction of Fas by progesterone prepares the cells to undergo apoptosis at the end of their function, which is determined by decrease in hormonal levels, as is the case in the endometrium if implantation fails to occur[37]. Thus, circulating estrogen and progesterone levels decrease and the endometrium is shed during a process called menstruation. Until electron microscope studies confirmed the presence of apoptotic bodies in human endometrial cells during the late secretory phase of the cycle, menstruation was regarded as ischaemic necrosis of the functional layer of the endometrium. Today we have enough evidence showing that during involution of the endometrium, endometrial cells undergo rapid regression via apoptosis in preparation for

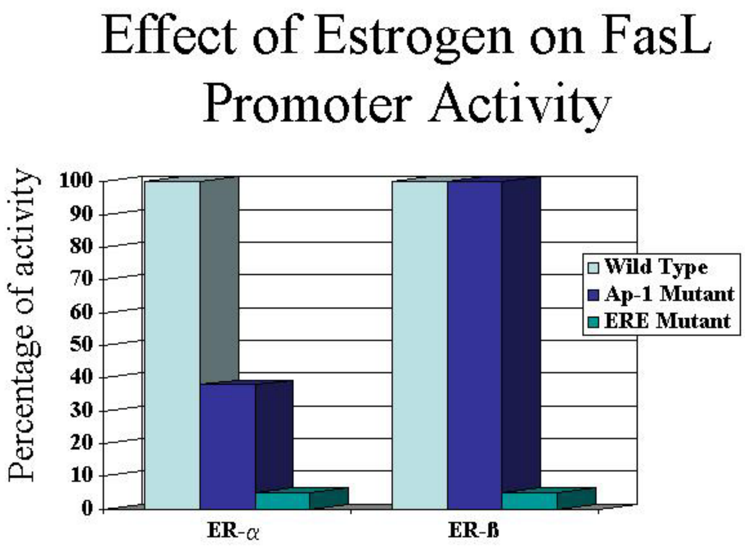

**FIGURE 3**. Effect of estrogen on FasL promoter activity. The effect of estrogen on FasL activity was determined using a luciferase reporter gene system. Both ERE and AP-1 motifs are necessary for estrogen-induced FasL activation in ERa bearing cells. On the other hand, only the ERE motif is necessary in ERβ bearing cells.

the next menstrual cycle. Part of this process is mediated by the Fas/FasL system[37]. We demonstrated the role of Fas/FasL in endometrial cells by showing that withdrawal of estrogen and progesterone activates the Fas pathway and induces cell death in endometrial cells *in vitro*. Additionally, blocking the Fas pathway with a competitive FasL antibody prevented apoptosis following estrogen and progesterone withdrawal[37].

The effects of estrogen and progesterone on the expression of FasL and Fas, respectively, exemplify the delicate balance existing between cell survival and cell death and the role sex hormones play in the regulation of this important apoptotic pathway.

## Mammary Gland

The normal development of mammary glands during pregnancy is characterized by a period of epithelial cell proliferation followed by the differentiation of milk-producing cells after parturition[38]. Walker and coworkers have demonstrated that in mouse and rat, mammary tissue involution is accompanied by the cleavage of chromatin into oligonucleotide fragments[39], a characteristic of apoptosis[1,40,41]. These and other studies[42,43] provide strong evidence that cell loss during involution occurs by programmed cell death. The occurrence of such remodeling is supported by experimental manipulations which have shown that after litter removal, lactation can be maintained and involution impeded by injection of lactant hormones[44,45].

In a recent study we demonstrated that although Fas protein is present during normal breast development, it is absent during pregnancy and lactation, only to return after weaning. On the other hand, FasL is present during pregnancy, lactation, and weaning but not in the virgin mouse. The overlapping expression of Fas and FasL during involution is accompanied by apoptosis of the mammary epithelium[3].

We further evaluated the role of Fas and FasL in mammary gland remodeling using the Fas deficient MRL/*lpr* mice, in which the Fas gene is interrupted by an early transposable element and carries a point mutation in the death domain, and the C3H/*gld* mice, which have a nonfunctional FasL[46,47]. Lack of Fas or functional FasL in the MRL/*lpr* and C3H/*gld* mice, respectively, prevented apoptosis of mammary cells during the first 3 days of involution. However, apoptotic cells were found at day 4 of involution, suggesting that the Fas/FasL system may play an important role in early stage of involution. The timing of the differences in mammary apoptotic cells in the MRL/*lpr* and C3H/*gld* mice is crucial in light of the two-stage model of involution proposed by Lund and coworkers[48]. The authors proposed that, postlactational involution of the mammary gland is characterized by two distinct phases. The first phase of involution is characterized by rapid induction of proapoptotic genes within the epithelium (days 1–4)[48,49]. This is the period when Fas and FasL are active based on the absence of apoptotic cells in the mammary glands of MRL/*lpr* and C3H/*gld* mice. The second phase of involution is characterized by the induction of genes encoding proteases within stroma cells that result in the remodeling of the gland[48]. This phase is Fas/FasL independent as shown by the presence of apoptotic cells in the mammary glands of MRL/*lpr* and C3H/*gld* mice. This chronology is consistent with Fas and FasL being expressed in the cell surface during the first phase resulting in the “suicide” of the secretory epithelium. Cells escaping the first phase are then removed by secondary mechanism that is Fas-independent. At this time, stroma cells, including macrophages, may induce cell death of the epithelium to ensure the removal of secretory cells[50].

The expression of Fas and FasL on the involuting mammary gland is not homogeneous, even in the same gland or duct. Using immunohistochemistry, we found that some cells were positively stained while other nearby cells were negative, suggesting differences in remodeling stages between cellular districts of the glands and ducts. Moreover, this heterogeneity of apoptosis provides a survival advantage since the entire function of the gland is not lost immediately when sucking stops. Rather, apoptotic factors (e.g., Fas/FasL) trigger graded programmed cell death and decreased milk production, allowing for sucking to be restarted if necessary. If pups resume suckling, the hormonal microenvironment, which constitutes a survival factor, inhibits apoptosis and promotes restoration of milk production. However, if suckling is not restored, the second phase of involution takes place, promoting disruption of basement membrane and extracellular matrix, resulting in a complete remodeling of the gland to a state resembling the mature virgin[3,49].

## Ovary

Two major stages of oocyte degeneration can be distinguished in the ovary: germ cell attrition, which occurs mainly prenatally, and follicle atresia, a process during reproductive life (reviewed in [51]). At 24 weeks of gestation, the number of oocytes in the human ovaries reaches a maximum of 7 million. At birth, about 0.7 to 2 million oocytes remain in the ovaries and at puberty only 400,000 are left. Additionally, during every estrous cycle, about 5–12 follicles resume development, and usually only 1 follicle becomes dominant and is ovulated, whereas the other follicles degenerate. Ovarian follicular degeneration or atresia is a hormonally controlled apoptotic process of the granulosa cells and to some extent the theca cells, which eliminates subordinate follicles[52]. Ovulation results in damage of the ovarian surface epithelium, which will be repaired by subsequent proliferation and apoptosis. After ovulation, the corpus luteum develops, which regresses after a few days in the case of nonpregnancy, or after a few months when successful pregnancy occurs. Several experiments suggest that there is an important role for the Fas-pathway in follicular atresia, ovarian surface epithelium repair and luteolysis.

The expression of Fas and FasL in mammalian ovaries varies between different cell types and throughout the menstrual cycle. Studies on mice ovaries revealed that granulosa cells of large follicles and some medium follicles express Fas, whereas no Fas expression was detected in granulosa cells of small follicles and in oocytes[53]. When we evaluated Fas and FasL expression in the female rat, FasL was highly expressed in estrous, decreased during metestrous and diestrous, and was not detected in proestrous, while Fas protein expression increased during proestrous and estrous and decreased during metestrous and diestrous[36]. In cows, Fas and FasL are expressed in granulosa and theca cells of day 5 and 11 follicles, however, the Fas mRNA level is higher in granulosa cells and the FasL mRNA level is higher in theca cells[54]. At day 5 of the estrous cycle, granulosa and theca cells of subordinate atretic follicles have higher Fas and FasL mRNA content than healthy dominant follicles, which correlates with increased sensitivity to FasL-induced apoptosis in granulosa, but not theca cells[54,55]. During transition of healthy dominant bovine follicles (day 5) into atretic dominant follicles (day 11), the level of Fas mRNA increases in theca cells, whereas the FasL mRNA level increases in granulosa cells[55]. Similarly, in human ovaries, Fas was localized to granulosa and theca cells of atretic, but not healthy antral follicles[56,57]. Therefore, it is likely that the Fas pathway is involved in the regression of subordinate follicles and follicle atresia.

Several studies also suggest that apoptosis during the regression of the corpus luteum (CL) may be mediated by the Fas-pathway. In mice, luteal cells of regressing CL have a marked increase in Fas expression compared to that of nonregressing CL[53]. Other evidence comes from studies performed on rat CL, where Fas and FasL were found in the cytoplasm of luteal cells, but not in endothelial cells[58]. When immunocytochemistry was performed on rat CL, Fas was detected only at day 1 of pregnancy and postpartum during luteolysis. Additionally, although FasL mRNA was present throughout pregnancy and postpartum, an increase in Fas-protein was found only during luteolysis[58].

*In vivo* experiments strengthened the *in vitro* experiments described above: intraperitoneal injection of an agonistic Fas antibody (RK-8) into mice resulted in the presence of more pycnotic bodies in granulosa cells of the follicles and a decrease in the number of ovulated ova and corpora lutea[53]. Additionally, 20-week old *lpr* and *gld* mutant mice, which have an extremely reduced Fas expression or encode a nonfunctional FasL protein, respectively, showed an increase in the number of medium follicles[53].

### Role of Gonadotrophic Hormones on Fas-Mediated Apoptosis

Ovarian cells are hormonal sensitive and respond to hormonal changes either by proliferating or dying. Numerous studies suggest a role of gonadotrophins in ovarian homeostasis. For example, gonadotrophin decreased the amount of apoptosis in rat granulosa cells and in primary cultures of human granulosa cells, which correlated with a decreased expression of both Fas and FasL[59,60].

In cycling rats, the prolactin surge during the proestrous afternoon induces apoptosis in luteal cells and correlates with an increase in FasL expression[61]. In another set of experiments, FasL mRNA was specifically found in CD3-positive luteal immunocytes, whereas Fas mRNA was only detected in the luteal steroidogenic cells[62]. Separation of the nonsteroidogenic from steroidogenic cells *in vitro* abolished prolactin-induced apoptosis, showing that the FasL-expressing immune cells are required for the prolactin-induced apoptosis of the steroidogenic cells[62]. However, during pregnancy, prolactin did not induce apoptosis in luteal steroidogenic cells, which was due to the action of progesterone[63]. Progesterone turned out to reduce the amount of Fas mRNA in the CL, and, thereby, suppressing the sensitivity of luteal steroidogenic cells to prolactin-induced apoptosis.

## MACROPHAGES, CLEARANCE, AND THE FAS/FASL SYSTEM

Macrophages are not merely scavengers of apoptotic cells, they are crucial both for the clearance of apoptotic cells, generated by tissue remodeling or injury, and for host defense against bacteria or protozoa. Where ingestion of bacteria triggers macrophages to secrete molecular mediators capable of inducing a protective, but potentially injurious inflammatory response[64], ingestion of apoptotic cells elicits the production of anti-inflammatory cytokines[65]. It was shown that the production of Th2-type cytokines by macrophages helps creating an anti-inflammatory microenvironment, which facilitates cell survival and proliferation and prevents tissue damage, and induces immune tolerance[65] .

Additionally, the lack of an inflammatory response during apoptosis has also been attributed to the rapid phagocytosis of apoptotic cells before cell lysis, thereby preventing the release of noxious contents which provoke inflammation and tissue damage[65]. Survival factors, such as estrogen, increase the phagocytic capacity of macrophages and, therefore, contribute to tissue survival[66].

For example, during the first few weeks of human implantation, a high number of macrophages is found in the maternal decidua and in tissues proximal to the placenta[67]. Similarly, macrophages accumulate at or near the implantation site in rodents[68]. Hunt and coworkers proposed that maternal macrophages assist in the tissue remodeling necessary to accommodate expansion of extraembryonic tissue[69]. Macrophages eliminate apoptotic uterine epithelial cells surrounding the blastocyst, during which an anti-inflammatory environment may be formed by increased anti-inflammatory cytokines[32] (Fig. 4).

The anti-inflammatory action of apoptotic cell phagocytosis may be perturbed in certain diseases ([32] and Fig. 5 ). For example, in the antiphospholipid syndrome, phospholipid antibodies in apoptotic cells bind to the Fc receptors in macrophages, resulting in secretion of TNF-a, a Th-1 type, proinflammatory cytokine[70]. If this occurs during pregnancy, cytokine production by macrophages and other cells at the maternal-fetal interface may be drastically altered[71]. Our studies with trophoblast cells are in concert with this concept, because they indicate that enhanced levels of proinflammatory macrophage products increase Fas expression and enhance trophoblast sensitivity to Fas-mediated apoptosis[32]. Two recent studies have described an increase in trophoblast cell apoptosis in pregnancies complicated by preeclampsia[72,73]. Interestingly, high levels of neutrophil activation have also been described in preeclamptic patients[74,75,76]. It is possible that a dense neutrophil infiltration may alter the surrounding environment at the maternal-fetal interface, thereby promoting the upregulation of Fas expression in nonimmune cells such as trophoblast and vascular endothelium and allowing FasL-induced inflammation and apoptosis[77].

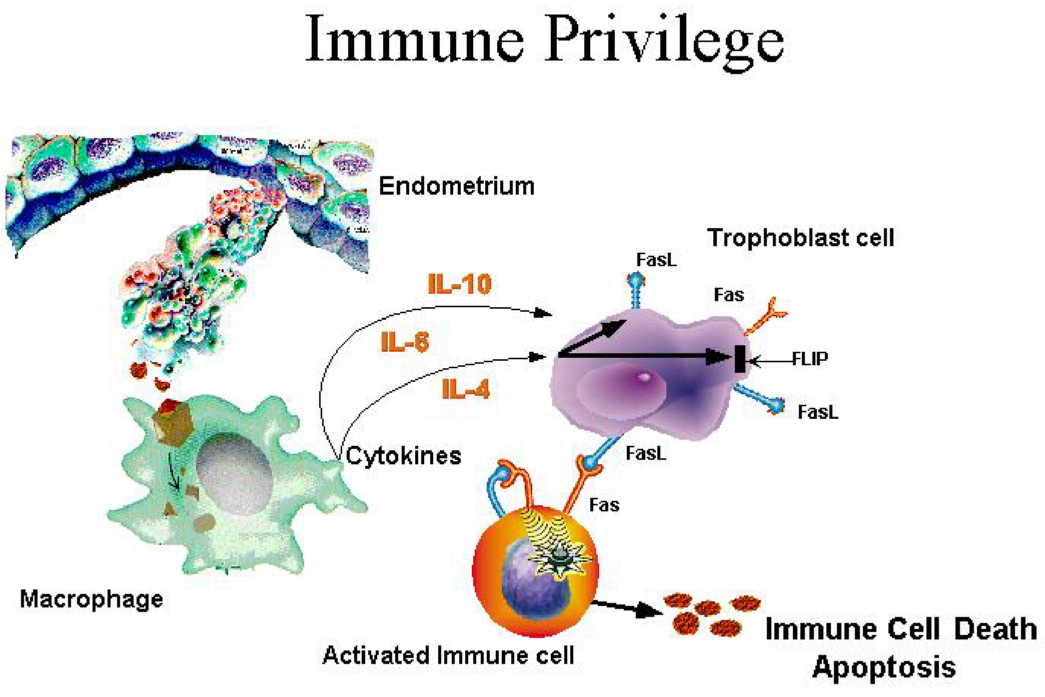

FIGURE 4A

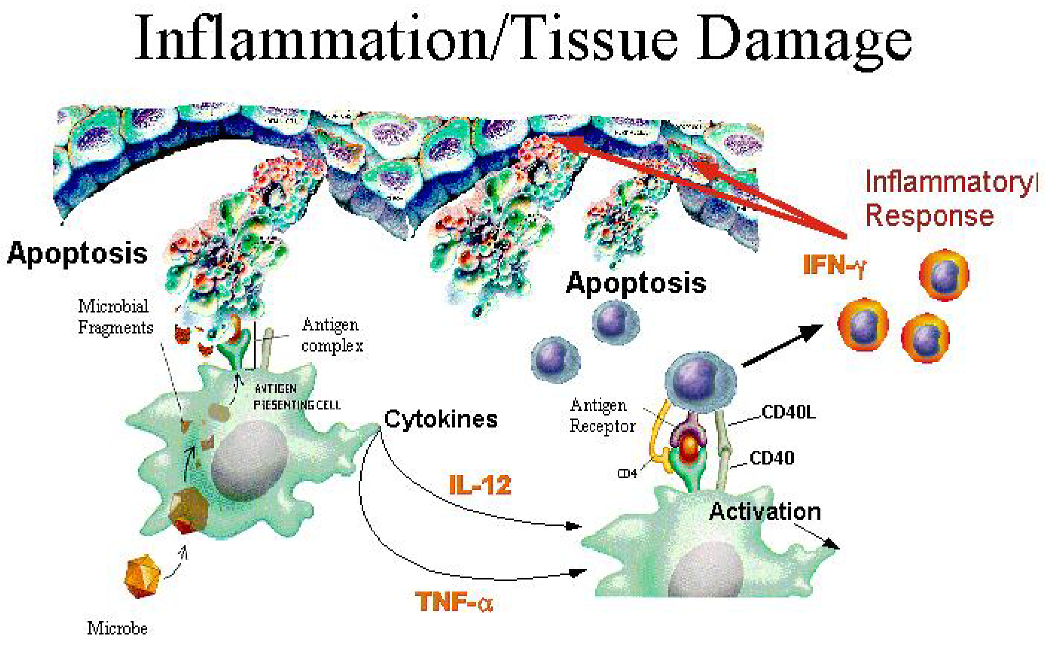

FIGURE 4B

**FIGURE 4**. Immune Privilege. A. Trophoblast cells induce apoptosis of activated immune cells through the expression of FasL on its surface. FasL expression is supported by locally produced anti-inflammatory cytokines. B. Bacterial infection or excessive apoptosis activates macrophages towards a proinflammatory cytokine profile. The inflammatory response further induces cell apoptosis and tissue damage.

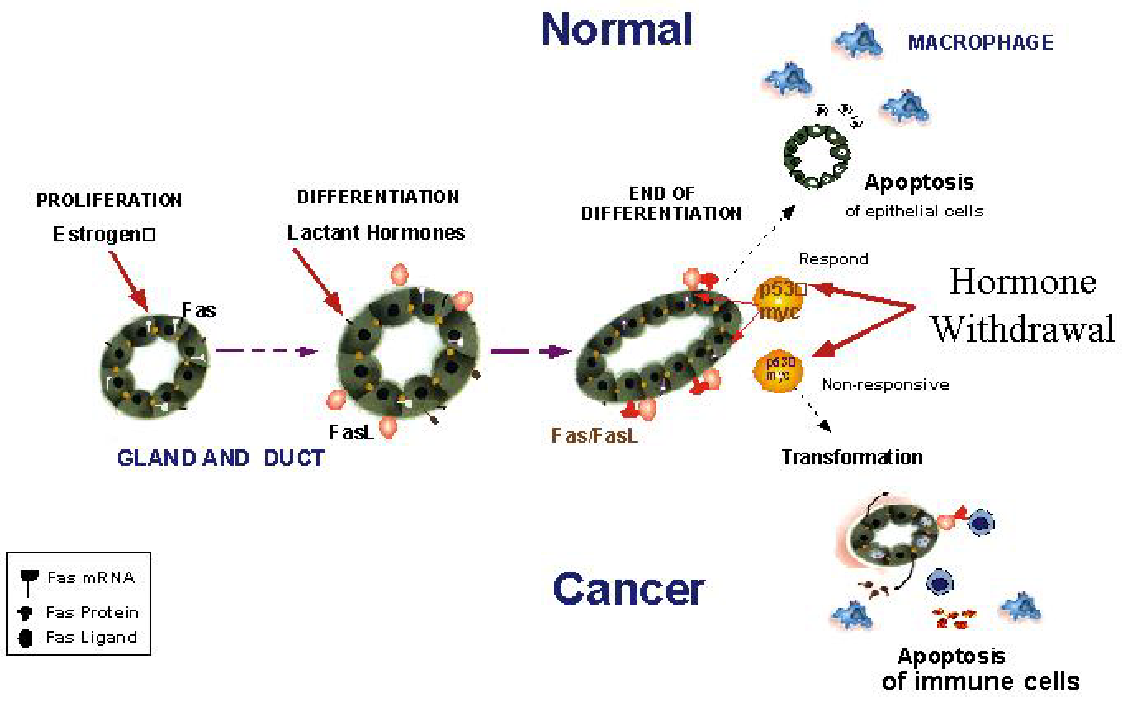

**FIGURE 5**. Model of tissue remodeling, normal and cancer.

## FAS AND FAS LIGAND EXPRESSION AND FUNCTION IN GYNECOLOGICAL CANCERS

Since Fas and FasL are expressed in reproductive tissues and, as discussed above, have an important role in tissue remodeling, aberrant alterations in the Fas or FasL expression may lead to abnormalities. Both Fas and FasL proteins are found in ovarian cancer tissue and primary ovarian cancer cultures[78,79]. Similarly, FasL expression was found in breast cancer cells[80,81] and its levels of expression have been shown to correlate with low prognostic value and decreased survival[82].

In ovarian carcinoma tissue, Fas mRNA levels, but not FasL mRNA levels, are decreased compared to normal ovarian tissue[83] Similarly, Mottolese and coinvestigators showed that breast cancer patients with Fas positive tumors exhibited longer disease-free survival than those having Fas-negative tumors[84].

Despite the presence of Fas and FasL, ovarian and breast cancer cells are resistant to Fas-mediated apoptosis[78,85].

## THE ROLE OF FAS LIGAND IN IMMUNE PRIVILEGE: THE GOOD AND THE BAD

The Fas/FasL system is involved in the establishment and maintenance of immune-privileged sites such as the eye, brain[86], testis, and trophoblast[87,88]. Immune privilege refers to organs or tissues that prohibit the spread of inflammation in response to an antigen in order to avoid the destruction of nearby tissue. Recent studies revealed that FasL expression in the eye limits inflammation by removing Fas-bearing immune cells that infiltrate the blood-ocular barrier. Similarly, in addition to the classical Brain Blood Barrier, we described the existence of an Immune Brain Barrier (IBB), which represents an active system regulating the movement of immune cells to and from the central nervous system. This IBB is generated by the expression of FasL on neurons and microglia cells in the neuropil and on astrocytes around blood vessels, functioning as an active and selective barrier to activated immune cells[36].

Tumors are also established as immune privileged sites since neoplastic cells are able to escape immune surveillance and avoid an inflammatory response. We and others have demonstrated that the expression of FasL in cancer cells may represent a mechanism by which neoplastic cells escape immune surveillance[81,89,90,91]. Cancer cells express FasL, which interacts with and activates the Fas receptor present on the surface of immune cells that infiltrate the tumor[81]. Furthermore, FasL expression on neoplastic cells is enhanced by cytokines and hormones produced by the immune infiltrate present in the vicinity of the tumor[92]. Besides reducing the number of circulating cytotoxic T cells, the expression of FasL on tumor cells can also abolish the production of antibodies and limit immunological memory against the tumor during a process called tumor counterattack[81,89].

In summary, it can be concluded that the Fas/FasL system mediates tissue remodeling and protects against the accumulation of malignant cells (the good); however, if the Fas pathway is blocked and the cell becomes resistant to Fas-mediated apoptosis, FasL in the malignant cell will induce apoptosis in the immune cells (the bad).

## THE MODEL

We can summarize the above-discussed studies in the following model using the mammary gland as an example of reproductive tissue (see Fig. 5). Thus, apoptosis of proliferating cells is prevented through the inhibition of Fas expression. Changes in the mammary gland microenvironment, such as hormonal withdrawal (e.g., postlactation), will deprive the cells of their support/survival stimuli, thereby allowing the activation of the apoptotic machinery through the Fas receptor. Tumor suppressor genes, such as p53 and c-myc, induce Fas expression and/or its translocation to the cell surface, where it meets its ligand and induces apoptosis. This process allows both normal tissue remodeling and removal of mutated cells (Fig. 5: Normal). However, a decrease in Fas expression/activation may lead to proliferation of transformed cells. Furthermore, FasL expression on neoplastic cells is enhanced by cytokines and hormones produced by the immune infiltrate present in the vicinity of the tumor. Cancer cells with an increased FasL expression might send apoptotic signals to the wrong cells, such as immune cells, and escape immune surveillance. Thereby an immune privileged site is created similar to those natural sites such as the eye, testis, and brain (Fig. 5: Cancer).

## ACKNOWLEDGMENT

This work was in part supported by Grants from the National Institutes of Health RO1 HD37137-01A2 and R01CA92435-01

## References

1. Kerr, J.F., Wyllie, A.H., and Currie, A.R. (1972) Apoptosis: a basic biological phenomenon with wide-ranging implications in tissue kinetics. *Br. J. Cancer* **26(4),** 239–257.
2. Wyllie, A., Kerr, J.F., and Currie, A. (1980) Cell death: the significance of apoptosis. *Int. Rev. Cytol*. **68**, 251–306.
3. Song, J. et al. (2000) Expression of Fas and Fas Ligand during pregnancy, lactation and involution and its potential role during mammary gland remodeling. *J. Clin. Invest.* **106(10),** 1209–1224.
4. Kerr, J.F., Wyllie, A.H., and Currie, A.R. (1972) Apoptosis: a basic biological phenomenon with wide-ranging implications in tissue kinetics. Br. J .Cancer **26(4),** 239–257.**[duplicate of #1]**

5. Krammer, P. (2000) CD95's deadly mission in the immune system. *Nature* **407**, 789–795.
6. Budihardjo, I. et al. (1999) Biochemical pathways of caspase activation during apoptosis. *Annu. Rev. Cell Dev. Biol*. **15,** 269–290.
7. McGahon A.J. et al. (1995) The end of the (cell) line: methods for the study of apoptosis in vitro. *Methods Cell Biol.* **46(1),** 53–85.
8. Hengartner, M.O. (2000) The biochemistry of apoptosis. *Nature* **407(6805),** 770–776.
9. Rich, T., Allen, R.L., and Wyllie, A.H. (2000) Defying death after DNA damage. *Nature* **407(6805),** 777–783.
10. Rich, T., Watson, C.J., and Wyllie, A. (1999) Apoptosis: the germs of death. *Nat. Cell Biol.* **1(3),** E69–71.
11. Nagata, S. and Golstein, P. (1995) The Fas death factor. *Science* **267**, 1449–1456.
12. Wallach, D. et al. (1999) Tumor necrosis factor receptor and Fas signaling mechanisms. *Annu. Rev. Immunol*. **17,** 331–367.
13. Stegh, A.H. et al. (1998) DEDD, a novel death effector domain-containing protein, targeted to the nucleolus. *EMBO J*. **17(20),** 5974–5986.
14. Schulze-Osthoff, K. et al. (1998) Apoptosis signaling by death receptors. *Eur. J. Biochem.* **254(3),** 439–459.
15. Schmitz, I. et al. (1999) Differences between CD95 type I and II cells detected with the CD95 ligand. *Cell Death Differ*. **6(9),** 821–822.
16. Muzio, M., Salvesen, G.S., and Dixit, V.M. (1997) FLICE induced apoptosis in a cell-free system. Cleavage of caspase zymogens. *J. Biol. Chem*. **272(5),** 2952–2956.
17. Scaffidi, C. et al. (2000) Analysis of the CD95 (APO-1/Fas) death-inducing signaling complex by high-resolution two-dimensional gel electrophoresis. *Methods Enzymol*. **322,** 363–373.
18. Scaffidi, C. et al. (1999) Apoptosis signaling in lymphocytes. *Curr. Opin. Immunol.* **11(3),** 277–285.
19. Brunner, T. et al. (1995) Cell-autonomous Fas (CD95)/Fas-ligand interaction mediates activation-induced apoptosis in T-cell hybridomas. *Nature* **373(6513),** 441–444.
20. Scaffidi, C. et al. (1999) Differential modulation of apoptosis sensitivity in CD95 type I and type II cells. *J. Biol. Chem*. **274(32),** 22532–22538.
21. Scaffidi, C., Krammer, P.H., and Peter, M.E. (1999) Isolation and analysis of components of CD95 (APO-1/Fas) death-inducing signaling complex. *Methods* **17(4),** 287–291.
22. Villunger, A. et al. (1999) Synergistic action of protein kinase C theta and calcineurin is sufficient for fas ligand expression and induction of a crmA-sensitive apoptosis pathway in jurkat T cells [In Process Citation]. Eur. J. Immunol. **29(11),** 3549–3561 [MEDLINE record in process].
23. Huang, D.C., Tschopp, J., and Strasser, A. (2000) Bcl-2 does not inhibit cell death induced by the physiological Fas ligand: implications for the existence of type I and type II cells. *Cell Death Differ*. **7(8),** 754–755.
24. Van Parijs, L., Peterson, D.A., and Abbas, A.K (1998) The Fas/Fas ligand pathway and Bcl-2 regulate T cell responses to model self and foreign antigens. *Immunity* **8(2),** 265–274.
25. Mor, G. et al. (2001) The role of the Fas/Fas ligand system in estrogen-induced thymic alteration. *Am. J. Reprod. Immunol*. **46(4),** 298–307.
26. Nagata, S. (1994) Fas and Fas ligand: a death factor and its receptor. *Adv. Immunol.* **57,** 129–135.
27. Takahashi, T. et al. (1994) Generalized lymphoproliferative disease in mice, caused by point mutation in the Fas ligand. *Cell* **76,** 969–976.
28. Sharma, K. et al. (2000) Death the Fas way: regulation and pathophysiology of CD95 and its ligand. *Pharmacol. Ther*. **88(3),** 333–347.
29. Yamashita, H. et al. (1999) Fas ligand, Fas antigen and Bcl-2 expression in human endometrium during the menstrual cycle. *Mol. Hum. Reprod*. **5(4),** 358–364.
30. Song, R.X. et al. (2001) Effect of long-term estrogen deprivation on apoptotic responses of breast cancer cells to 17beta-estradiol. *J. Natl. Cancer Inst*. **93(22),** 1714–1723.
31. Mor, G. et al. (2000) Regulation of Fas ligand expression in breast cancer cells by estrogen: functional differences between estradiol and tamoxifen. *J. Steroid Biochem. Mol. Biol.* **73(5),** 185–194.
32. Aschkenazi, S. et al. (2002) Differential regulation and function of the Fas/Fas Ligand system in human trophoblast cells. *Biol. Reprod*., in press.
33. Matsui, K. et al. (1997) Proteasome regulation of Fas ligand cytotoxicity. *Eur. J. Immunol.* **27(9),** 2269–2278.
34. Nilsen, J., Mor, G., and Naftolin, F. (2000) Estrogen-regulated developmental neuronal apoptosis is determined by estrogen receptor subtype and the Fas/Fas ligand system. *J. Neurobiol*. **43(1),** 64–78.
35. Sapi, E. et al. (2002) Regulation of Fas Ligand expression by estrogen in normal ovary. *J. Soc. Gynecol. Invest*. **9(4).**
36. Rutherford, T. et al. (2000) Absence of estrogen receptor ß expression in metastatic ovarian cancer. *J. Obstet. Gynecol*. **96(3),** 417–421.
37. Song, J. et al. (2002) Hormonal regulation of Fas and FasL expression and apoptosis in the normal human endometrium. *Mol. Hum. Reprod*. **8**(5), 447–455.
38. Russo, J., Tay, L.K., and Russo, I.H. (1982) Differentiation of the mammary gland and susceptibility to carcinogenesis. *Breast Cancer Res. Treat*. **2(1),** 5–73.
39. Walker, N.I., Bennett, R.E., and Kerr, J.F. (1989) Cell death by apoptosis during involution of the lactating breast in mice and rats. *Am. J. Anat*. **185(1),** 19–32.

40. Morris, R.G. et al. (1984) Hormone-induced cell death. 2. Surface changes in thymocytes undergoing apoptosis. *Am. J. Pathol*. **115(3),** 426–436.
41. Duvall, E., Wyllie, A.H., and Morris, R.G. (1985) Macrophage recognition of cells undergoing programmed cell death. *Immunology* **56(2),** 351–358.
42. Quarrie, L.H., Addey, C.V., and Wilde, C.J. (1995) Apoptosis in lactating and involuting mouse mammary tissue demonstrated by nick-end DNA labelling. *Cell Tissue Res.* **281(3),** 413–419.
43. Quarrie, L.H., Addey, C.V., and Wilde, C.J. (1996) Programmed cell death during mammary tissue involution induced by weaning, litter removal, and milk stasis. *J. Cell Physiol.* **168(3),** 559–569.
44. Ossowski, L., Biegel, D., and Reich, E. (1979) Mammary plasminogen activator: correlation with involution, hormonal modulation and comparison between normal and neoplastic tissue. *Cell* **16(4),** 929–940.
45. Strange, R. et al. (1995) Programmed cell death during mammary gland involution. *Methods Cell Biol*. **46,** 355–368.
46. Adachi, M. et al. (1995) Targeted mutation in the Fas gene causes hyperplasia in peripheral lymphoid organs and liver. *Nat. Genet.* **11(3),** 294–300.
47. Adachi, M., Watanabe-Fukunaga, R., and Nagata, S. (1993) Aberrant transcription caused by the insertion of an early transposable element in an intron of the Fas antigen gene of lpr mice. *Proc. Natl. Acad. Sci. U. S. A.* **90(5),** 1756–1760.
48. Lund, L.R. et al. (1996) Two distinct phases of apoptosis in mammary gland involution: proteinase-independent and -dependent pathways. *Development* **122(1),** 181–193.
49. Li, M. et al. (1997) Mammary-derived signals activate programmed cell death during the first stage of mammary gland involution. *Proc. Natl. Acad. Sci. U. S. A.* **94(7),** 3425–3430.
50. Furth, P.A. (1999) Introduction: mammary gland involution and apoptosis of mammary epithelial cells. *J. Mammary Gland Biol. Neoplasia* **4(2),** 123–127.
51. Kaipia, A. and Hsueh, A.J. (1997) Regulation of ovarian follicle atresia. *Annu. Rev. Physiol.* **59,** 349–363.
52. Hughes, F.M., Jr. and Gorospe, W.C. (1991) Biochemical identification of apoptosis (programmed cell death) in granulosa cells: evidence for a potential mechanism underlying follicular atresia. *Endocrinology* **129(5)**, 2415–2422.
53. Sakamaki, K. et al. (1997) Involvement of Fas antigen in ovarian follicular atresia and luteolysis. *Mol. Reprod. Dev*. **47(1**), 11–18.
54. Porter, D.A. et al. (2000) Expression and function of Fas antigen vary in bovine granulosa and theca cells during ovarian follicular development and atresia. *Biol. Reprod.* **62(1),** 62–66.
55. Porter, D.A. et al. (2001) Relationship of Fas ligand expression and atresia during bovine follicle development. *Reproduction* **121(4),** 561–566.
56. Kondo, H. et al. (1996) Immunological evidence for the expression of the Fas antigen in the infant and adult human ovary during follicular regression and atresia. *J. Clin. Endocrinol. Metab.* **81(7),** 2702–2710.
57. Maruo, T. et al. (1999) Regulation of granulosa cell proliferation and apoptosis during follicular development. *Gynecol. Endocrinol*. **13(6),** 410–419.
58. Roughton, S.A. et al. (1999) Fas and Fas ligand messenger ribonucleic acid and protein expression in the rat corpus luteum during apoptosis-mediated luteolysis. *Biol. Reprod.* **60(4),** 797–804.
59. Kim, J.M., Yoon, Y.D., and Tsang, B.K. (1999) Involvement of the Fas/Fas ligand system in p53-mediated granulosa cell apoptosis during follicular development and atresia. *Endocrinology* **140(5),** 2307–2317.
60. Asselin, E. et al. (2000) Mammalian follicular development and atresia: role of apoptosis. *Biol. Signals Recept.* **9(2),** 87–95.
61. Kuranaga, E. et al. (1999) Fas/Fas ligand system in prolactin-induced apoptosis in rat corpus luteum: possible role of luteal immune cells. *Biochem. Biophys. Res. Commun*. **260(1),** 167–173.
62. Kuranaga, E. et al. (2000) Requirement of the Fas ligand-expressing luteal immune cells for regression of corpus luteum. *FEBS Lett*. **472(1),** 137–142.
63. Kuranaga, E. et al. (2000) Progesterone is a cell death suppressor that downregulates Fas expression in rat corpus luteum. *FEBS Lett*. **466(2-3)**, 279–282.
64. Medzhitov, R. and Janeway, C., Jr. (2000) *Innate immunity. N. Engl. J. Med.* **343(5),** 338–344.
65. Voll, R.E. et al. (1997) Immunosuppressive effects of apoptotic cells. *Nature* **390(6658),** 350–351.
66. Schreiber, A. et al. (1988) Effect of endogenous and synthetic sex steroids on the clearance of antibody-coated cells. *J. Immunol*. **141(9),** 2959–2966.
67. Miller, L. and Hunt, J. (1996) Sex steroids hormones and macrophage function. *Life Sci*. **59(1),** 1–14.
68. Tachi, C. and Tachi, S. (1989) Role of macrophages in the maternal recognition of pregnancy. *J. Reprod. Fertil. Suppl*. **37,** 63–68.
69. Hunt, J. (1989) Cytokine networks in the uteroplacental unit: macrophages as pivotal regulatory cells. *J. Reprod. Immunol*. **16,** 1–17.
70. Manfredi, A.A. et al. (1998) Apoptotic cell clearance in systemic lupus erythematosus. I. Opsonization by antiphospholipid antibodies. *Arthritis Rheum*. **41(2)**, 205–214.
71. Guleria, I. and Pollard, J.W. (2000) The trophoblast is a component of the innate immune system during pregnancy. *Nat. Med*. **6(5),** 589–593.

72. Fisher, D. (1994) Apoptosis in cancer therapy. *Cell* **78,** 539–542.
73. Allaire, A. et al. (2000) Placental apoptosis in preeclampsia. *Obstet. Gynecol*. **96(2),** 271–276.
74. Clark, P., Boswell, F., and Greer, I.A. (1998) The neutrophil and preeclampsia. *Semin. Reprod. Endocrinol*. **16(1),** 57–64.
75. Greer, I.A. et al. (1991) Neutrophil activation is confined to the maternal circulation in pregnancy-induced hypertension. *Obstet. Gynecol*. **78(1),** 28–32.
76. Greer, I.A. et al. (1989) Neutrophil activation in pregnancy-induced hypertension. *Br. J. Obstet. Gynaecol*. **96(8),** 978–982.
77. Varani, J. et al. (1992) Human umbilical vein endothelial cell killing by activated neutrophils. Loss of sensitivity to injury is accompanied by decreased iron content during in vitro culture and is restored with exogenous iron. *Lab. Invest*. **66(6),** 708–714.
78. Baldwin, R.L., Tran, H., and Karlan, B.Y. (1999) Primary ovarian cancer cultures are resistant to Fas-mediated apoptosis. *Gynecol. Oncol*. **74(2),** 265–271.
79. Munakata, S. et al. (2000) Expressions of Fas ligand and other apoptosis-related genes and their prognostic significance in epithelial ovarian neoplasms. *Br. J. Cancer* **82(8),** 1446–1452.
80. Xerri, L. et al. (1997) Fas ligand is not only expressed in immune privileged human organs but is also coexpressed with Fas in various epithelial tissues. *Mol. Pathol.* **50(2),** 87–91.
81. Gutierrez, L. et al. (1999) The Fas/Fas-Ligand system: a mechanism for immune evasion in human breast carcinomas. Breast Cancer Res. Treat. **54(3),** 245–253.
82. Reimer, T. et al. (2000) FasL:Fas ratio—a prognostic factor in breast carcinomas. *Cancer Res*. **60(4),** 822–828.
83. Das, H. et al. (2000) Quantitation of Fas and Fas ligand gene expression in human ovarian, cervical and endometrial carcinomas using real-time quantitative RT-PCR. *Br. J. Cancer* **82(10),** 1682–1688.
84. Mottolese, M. et al. (2000) Prognostic relevance of altered Fas (CD95)-system in human breast cancer. *Int. J. Cancer* **89(2),** 127–132.
85. Keane, M.M. et al. (1996) Fas expression and function in normal and malignant breast cell lines. *Cancer Res*. **56(20),** 4791–4798.
86. Bechmann, I. et al. (1999) FasL (CD95L, Apo1L) is expressed in the normal rat and human brain-evidence for the existence of an immunological brain barrier. *Glia* **27,** 62–74.
87. Bamberger, A. et al. (1997) Expression of the apoptosis-inducing Fas Ligand (FasL) in human first and third trimester placenta and choriocarcinoma cells. *J. Clin. Endocrinol. Metab.* **82(9),** 3173–3175.
88. Mor, G. et al. (1998) Fas-Fas ligand system induced apoptosis in human placenta and gestational trophoblastic disease. *Am. J. Reprod. Immunol*. **40,** 89–95.
89. O'Connell, J. et al. (1996) The Fas counter attack: Fas mediated T cell killing by colon cancer cells expressing Fas ligand. *J. Exp. Med*. **184,** 1075–1082.
90. O'Connell, J. et al. (2000) Interferon-gamma sensitizes colonic epithelial cell lines to physiological and therapeutic inducers of colonocyte apoptosis. *J. Cell Physiol*. **185(3),** 331–338.
91. O'Connell, J. et al. (2001) Immune privilege or inflammation? Insights into the Fas ligand enigma. *Nat. Med*. **7(3),** 271–274.
92. Mor, G. et al.(1998) Macrophages, estrogen and the microenvironment of breast cancer. *J. Steroid Biochem. Mol. Biol*. **67(5-6),** 403–411.
